# Mg Plating in Nonethereal Electrolyte Solutions: Fact or Misconception?

**DOI:** 10.1002/cssc.202500418

**Published:** 2025-05-04

**Authors:** Toshihiko Mandai

**Affiliations:** ^1^ Functional Electrolyte Synthesis Team Research Center for Energy and Environmental Materials (GREEN) National Institute for Materials Science (NIMS) 1‐1 Namiki Tsukuba Ibaraki 305‐0044 Japan

**Keywords:** decompositions, Mg plating, nonethereal electrolyte solutions

## Abstract

Rechargeable Mg metal batteries (RMMBs) have garnered significant attention due to their outstanding electrochemical energy storage performance. Ether solvents are widely used as a primary component of RMMB electrolytes because of their excellent compatibility with Mg metal. However, the poor anodic stability of ethers hinders the development of high‐energy‐density RMMBs. To address this limitation, researchers have explored novel electrolyte formulations based on nonethereal solvents. However, the interpretation of reported results remains controversial due to a lack of solid experimental evidence confirming Mg plating reactions in such media. This article provides an overview of Mg electrochemistry in both ethereal and nonethereal electrolyte systems. Comprehensive cyclic voltammetry studies on the electrochemical Mg plating/stripping activity in various electrolyte systems reaffirm that only a limited range of solvents—including ethers, amines, and dialkylsulfones—support reversible Mg plating/stripping. In contrast, Mg plating is rarely observed in nitrile, amide, carbonate, and phosphate electrolytes, despite voltammograms exhibiting seemingly reversible responses. These findings caution researchers against assuming voltammetric responses correspond to Mg plating/stripping without solid evidence of Mg deposits. Finally, a protocol for assessing whether proposed (uncommon) electrolyte systems genuinely support Mg plating/stripping is presented.

## Graphical abstract

An overview of Mg electrochemistry in both ethereal and nonethereal electrolyte systems was provided. Comprehensive reviewing on the electrochemical Mg plating/stripping activity reaffirms that only a limited range of solvents support reversible Mg plating/stripping. A protocol for assessing whether proposed electrolyte systems genuinely support Mg plating/stripping is also presented.

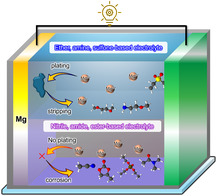

## 1 Introduction

With advancements in science and technology, the demand for electricity continues to rise. According to a report published by the International Energy Agency in January 2024, electricity consumption is increasing due to factors such as the expansion of artificial intelligence. Global electricity demand is projected to reach 1000 TWh in 2026, doubling that of 2022.^[^ ^1^ ^]^ In 2022, thermal power generation from fossil fuels accounted for more than 70% of total electricity production, leading to significant greenhouse gas emissions and environmental degradation. Therefore, the development of environmentally benign energy storage and grid‐balancing technologies based on renewable energy sources is crucial for achieving a sustainable society. Rechargeable batteries utilizing materials free from resource constraints, high production costs, and geopolitical risks are among the most promising energy storage technologies. Among potential candidates, rechargeable Mg metal batteries (RMMBs) have attracted considerable attention due to their excellent electrochemical energy‐storage performance. By leveraging the low electrode potential of Mg, its divalent nature, and the resulting high storage capacity, RMMBs have the potential to achieve energy densities comparable to those of current lithium‐ion batteries while benefiting from lower material costs.^[^ ^2^ ^]^

Despite their promising potential as energy storage devices, RMMBs remain in the early stages of development. Due to the sluggish Mg^2+^ diffusion kinetics in oxide‐based crystalline lattices, research has focused on alternative positive electrode materials, such as chalcogenides and redox‐active porous organic materials.^[^ ^3^, ^4^, ^5^ ^]^ Recent efforts to design oxide‐based materials with well‐structured Mg^2+^ diffusion pathways and optimized particle morphologies have led to improvements in Mg^2+^ storage performance, unlocking the potential of certain oxide‐based materials.^[^ ^6^, ^7^, ^8^ ^]^ However, the development of suitable electrolytes for RMMBs remains a critical challenge, as conventional lithium‐ion battery electrolytes induce severe passivation of Mg metal.^[^ ^9^ ^]^ Electrolytes play a crucial role not only in transporting carrier ions between electrodes but also as reaction media at electrode interfaces. In RMMBs, electrolyte solutions must enable reversible electrochemical reactions at both the Mg metal and the selected positive electrodes without causing detrimental side reactions. It is widely recognized in the RMMB research community that ether‐based solvents are essential electrolyte components due to their excellent chemical and electrochemical stability with Mg electrodes. However, the highly reactive nature of Mg leads to undesired reactions with electrolyte components, forming passivation layers composed of magnesium oxides, hydroxides, fluorides, and other insulating species that hinder Mg^2+^ migration at the interface.^[^ ^9^, ^10^ ^]^ The characteristics of these passivation layers differ significantly from that of the conventional solid electrolyte interphase (SEI) found in alkali metal‐based batteries. Therefore, the development of electrolyte materials that remains inert toward Mg metal while allowing efficient electrochemical Mg plating and stripping is essential for advancing RMMBs.

As described above, ether solvents are essential primary components of RMMB electrolytes due to their excellent compatibility with Mg metal. However, a critical challenge hindering the practical implementation of RMMBs also stems from the chemical nature of ether solvents. Their low oxidative stability limits the efficient operation of high‐energy‐density batteries.^[^ ^11^ ^]^ In certain lithium and sodium battery electrolyte systems—such as highly concentrated or localized highly concentrated electrolytes—regulating the solvation shell has been shown to improve the oxidative stability of ethers through the electric‐field effects of Lewis acidic metal ions.^[^ ^12^, ^13^, ^14^ ^]^ However, this solvation shell modulation approach compromises the reductive stability of strongly bound ether solvents due to collective shifts in ionization potential and electron affinity upon solvation, leading to inferior compatibility with the highly reductive Mg metal negative electrode.^[^ ^15^, ^16^ ^]^ Partial fluorination of the ether structure is a double‐edged sword: while the introduction of electron‐withdrawing groups enhances anodic stability, it simultaneously degrades cathodic stability.^[^ ^17^, ^18^ ^]^ Additionally, the low solubility of conventional Mg salts in most organic solvents presents another challenge in achieving highly concentrated electrolyte solutions. Furthermore, the autoxidation of ethers during long‐term storage poses serious safety concerns.

To address these challenges, researchers have investigated innovative electrolyte formulations based on nonethereal solvents. The use of oxidatively stable solvents, such as carbonates and sulfones—commonly employed in high‐voltage lithium batteries—offers a potential pathway for realizing high‐energy‐density RMMBs. Reports have claimed successful battery cycling of prototype cells using these nonethereal electrolyte solutions in combination with rationally designed functional interfaces.^[^ ^19^, ^20^ ^]^ These findings suggest the possibility of groundbreaking advancements in the RMMB research field. However, the interpretation of these results remains controversial due to the lack of solid experimental evidence confirming Mg plating reactions in such media, as well as the absence of clear validation methodologies. This uncertainty may lead to misconceptions about the intrinsic potential of nonethereal electrolyte systems, resulting in misdirected research efforts and ultimately delaying the realization of practical RMMBs.

**Figure 1:**
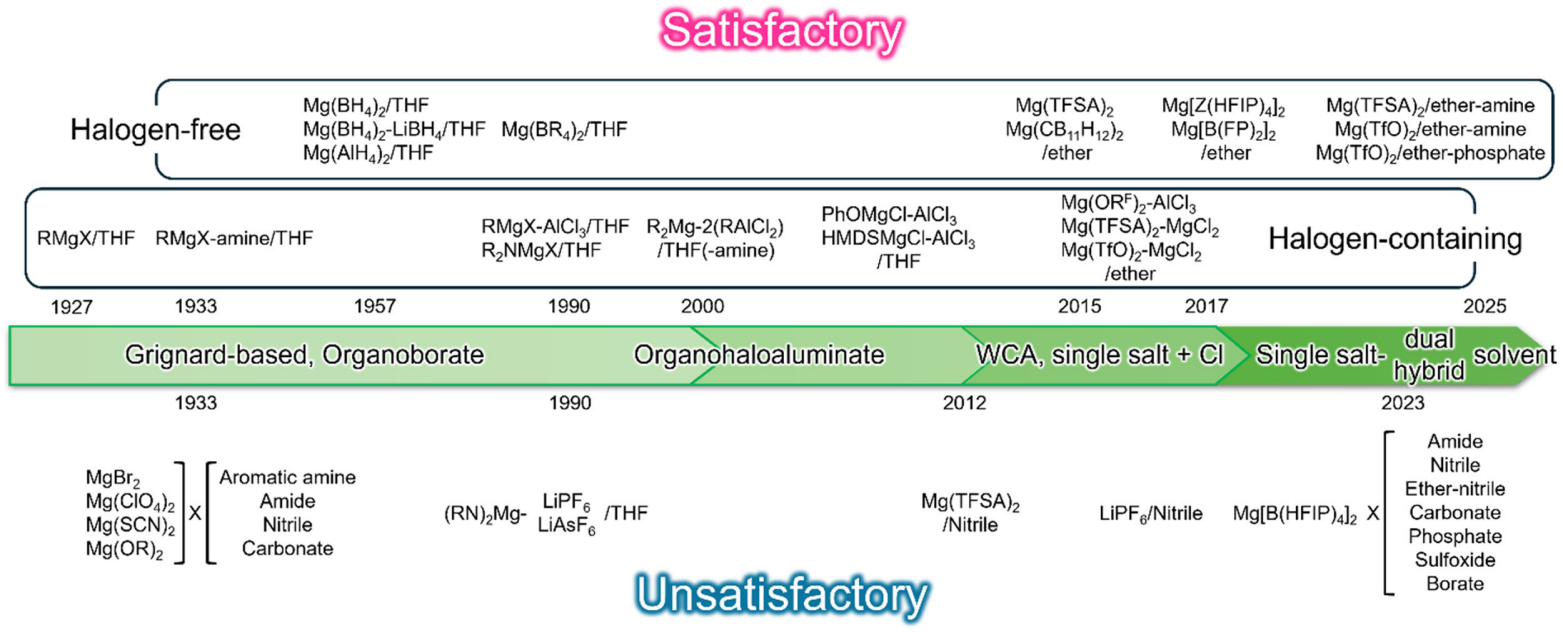
Overview of representative electrolyte solutions based on the Mg plating activity.

A summary of representative electrolyte solutions is presented in **Figure** **1**. In this article, we review the fundamental understanding of Mg electrochemistry in ethereal solutions and outline the characteristics of nonethereal electrolyte systems with respect to their formulations. Additionally, a proposed experimental protocol for evaluating Mg plating in nonethereal solutions is introduced as a guideline for assessing the electrochemical activity of unconventional electrolyte systems. It should be emphasized that the purpose of this article is not to criticize previous studies but to disseminate the collective knowledge of earlier research to the broader RMMB community, fostering a constructive and informed research environment.

**Scheme 1:**
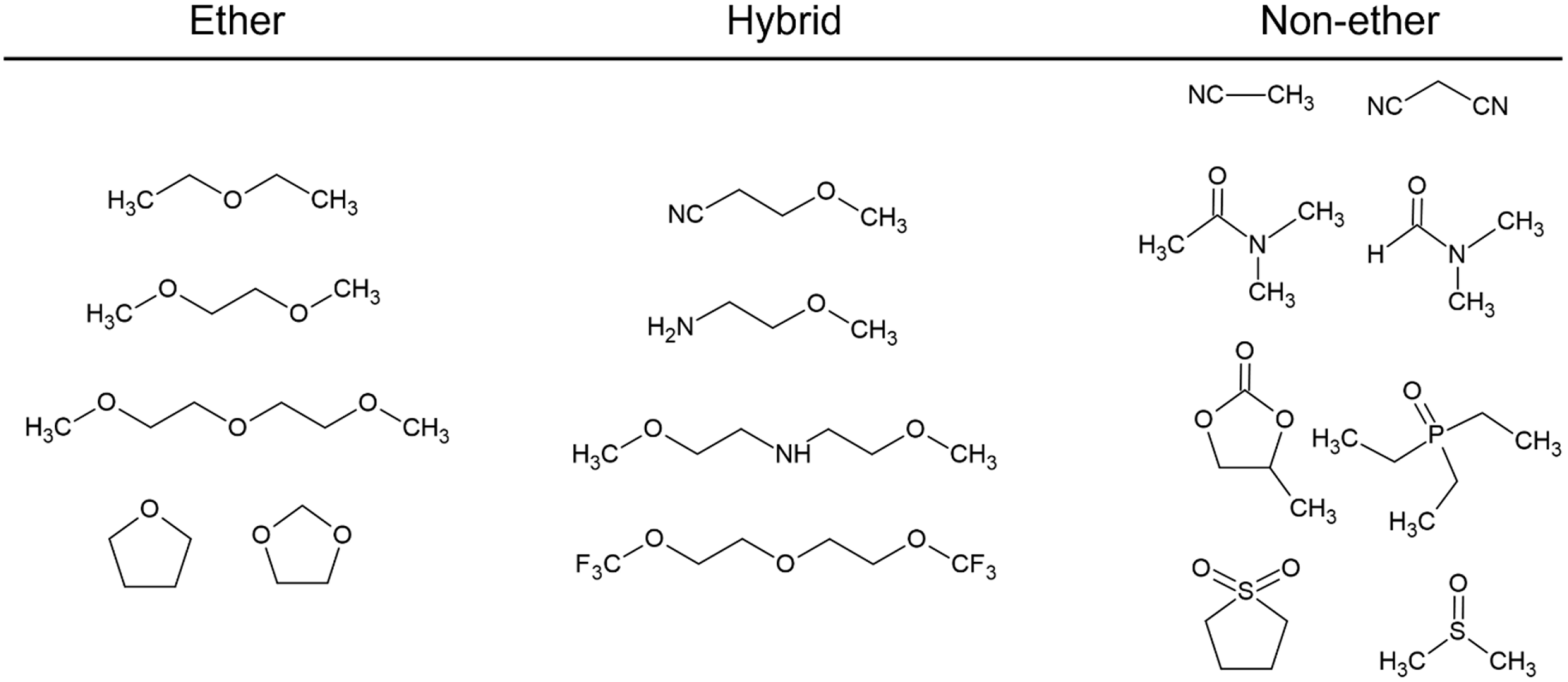
Category of electrolyte solvents in this article.

Note that, in this article, ether solvents are defined as compounds containing a C—O—C unit in their molecular structure, regardless of conventional nomenclature. In this context, alkylalkoxyamine compounds, for instance, are classified as ether solvents or as intramolecular amine‐ether hybrid systems (**Scheme** **1**).

## 2 Mg Electrochemistry in Ethereal Solutions

### 2.1 General Understandings of Mg Electrochemistry

Revisiting Mg electrochemistry in conventional ethereal solutions provides essential background knowledge. Reversible Mg plating/stripping is a fundamental electrode reaction at the negative electrode in RMMBs. While alkali metals can be plated and stripped in a variety of electrolyte formulations, this is not the case for Mg. Attempts to achieve reversible Mg plating/stripping in nonethereal, carbonyl compound‐based solutions, including carbonates and amides, have been unsuccessful for nearly a century.^[^ ^21^ ^]^ In contrast, Mg plating (electrolysis) from certain Grignard reagents and their combinations with alkylamines was observed as early as the same period.^[^ ^21^, ^22^ ^]^ The concept of combining amines (alkoxyamines) with ether solvents for solvation sheath regulation has recently been revisited (vide infra).^[^ ^23^, ^24^, ^25^, ^26^, ^27^, ^28^ ^]^ Additionally, Mg plating in halide‐free ethereal solutions of complex borohydrides, such as Mg(BH_4_)_2_ and aluminum hydrides like Mg(AlH_4_)_2_, was reported by Connor et al. in 1953.^[^ ^29^ ^]^ A pioneering comprehensive study on Mg plating/stripping in ethereal electrolyte solutions was later provided by Gregory et al. in 1990.^[^ ^30^ ^]^ That study systematically examined the effects of organomagnesium halide (RMgX) structural modifications, the integration of Lewis acid AlCl_3_, the compatibility of various anions with Mg metal, and the electrochemical behavior of organoborate‐based electrolytes. The first two approaches were later revisited to enhance the electrochemical performance of RMgX‐ and organoborate‐based ethereal solutions. Furthermore, the poor compatibility of hexafluorophosphate (PF_6_^−^) and hexafluoroarsenate (AsF_6_^−^) due to Mg passivation via undesired side reactions had already been identified in the preceding work,^[^ ^30^ ^]^ highlighting the importance of thorough literature review to prevent redundant efforts by subsequent researchers. This pioneering work also reported electrochemical Mg^2+^ intercalation in various oxide and sulfide‐based frameworks with a conventional cell set‐up using Mg metal negative electrode.^[^ ^30^ ^]^ Followed by this ground‐breaking achievement, Aurbach et al. achieved the first prototype of RMMBs using the Chevrel phase Mo_6_S_8_ as a positive electrode, ethereal solution of magnesium organohalualuminate compounds as an electrolyte, and Mg metal as a negative electrode in 2000.^[^ ^31^ ^]^

The mechanism of Mg plating/stripping in ethereal solutions strongly depends on the electrolyte constituents. In solutions containing halide anions, the mechanism is particularly complex due to the Schlenk equilibrium and halide‐bridging complex formation, which arise from the unique binding characteristics of halides. Such complex formation in halide‐containing ethereal solutions has been confirmed through X‐ray crystallography, vibrational spectroscopy, mass spectrometry, and theoretical calculations, independent of the Mg source. Indeed, Cl‐bridged complexes have been characterized in single crystals grown from a variety of ethereal solutions, including RMgCl‐AlCl_3_,^[^ ^32^ ^]^ MgCl(HMDS)‐AlCl_3_,^[^ ^33^ ^]^ PhMgCl‐B(Mes)_3_,^[^ ^34^ ^]^ Mg(TFSI)_2_‐AlCl_3_‐MgCl_2_,^[^ ^35^ ^]^ Mg(TFSI)_2_‐MgCl_2_,^[^ ^36^ ^]^ ROMgCl‐AlCl_3_,^[^ ^37^ ^]^ and Mg(FP)_2_‐AlCl_3_^[^ ^38^ ^]^ where HMDS, B(Mes)_3_, TFSI, and FP refer to hexamethyldisilazide, tris(3,5‐dimethylphenyl)borate, bis(trifluoromethanesulfonyl)imide, and perfluorinated pinacolate, respectively. This evidence suggests that the species responsible for electrochemical Mg plating/stripping activity in halide‐containing ethereal electrolytes are such complexes, and the compatibility of the remaining species with Mg determines the reaction efficiency. The relatively poor Mg plating/stripping efficiency of the LiPF_6_‐MgCl_2_ electrolyte further supports this hypothesis, as PF_6_‐containing electrolyte solutions are likely nonviable due to the poor compatibility of this anion with Mg.^[^ ^39^ ^]^ The electrochemical activity of this system is likely initiated by the suppression of severe passivation layer formation via the etching or shielding effects of adsorbed Cl^−^ species.^[^ ^40^, ^41^ ^]^ A detailed investigation of the role of Cl^−^ species in electrochemical Mg plating/stripping is beyond the scope of this study.

Despite the excellent Mg plating/stripping characteristics of halide‐containing ethereal electrolyte solutions, halide‐free electrolytes are highly desirable for practical applications due to the severe corrosion issues caused by halide species on cell components.^[^ ^42^, ^43^ ^]^ Additionally, the limited anodic stability of Cl^−^ in electrolyte solutions (2Cl^−^ → Cl_2_ + 2e; 1.36 V vs. SHE, ≈3.7 V vs. Mg^2+^/Mg) restricts the realization of high‐voltage RMMBs. Ethereal solutions of complex hydrides and magnesium organoborates represent the first generation of halide‐free electrolytes capable of supporting reversible Mg plating/stripping.^[^ ^29^, ^30^ ^]^ Due to the weak coordination ability of tetra‐substituted bulky organoborate anions, Mg^2+^ and the counter organoborate anions remain dissociated in ethereal solutions, requiring Mg^2+^ to be solvated by ether solvents for sufficient stabilization. The relatively high ionic conductivities of these solutions reflect the dissociative nature of magnesium organoborates.^[^ ^30^, ^44^ ^]^ Mg plating/stripping in such electrolytes involves desolvation and dissociation at the electrode–electrolyte interface, followed by charge transfer.

**Figure 2:**
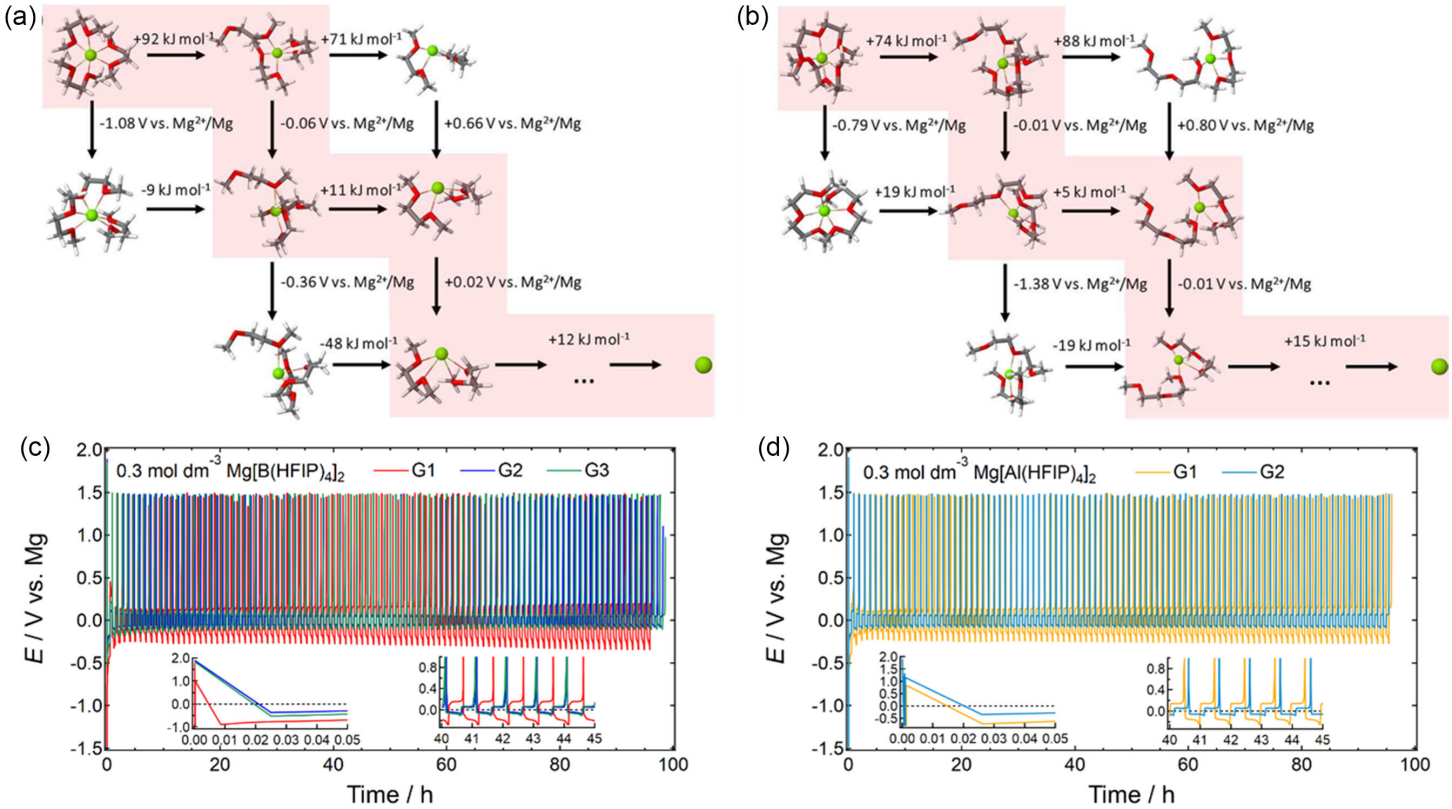
Possible desolvation (horizontal) and reduction (vertical) processes for a) [Mg(G1)_3_]^2+^ and b) [Mg(G2)_2_]^2+^. Reproduced with permission.^[^ ^47^ ^]^ Copyright 2021, Wiley‐VCH. Galvanostatic magnesium deposition–dissolution cycling profiles for [Mg | Cu] cells fabricated using c) 0.3 mol dm^−3^ Mg[B(HFIP)_4_]_2_/G*n* (*n* = 1 − 3) and d) 0.3 mol dm^−3^ Mg[Al(HFIP)_4_]_2_/G*n* (*n* = 1, 2) electrolytes. Reproduced with permission.^[^ ^48^ ^]^ Copyright 2021, The Royal Society of Chemistry.

Since the desolvation/dissociation process at the interface is considered the rate‐determining step for alkali metal plating,^[^ ^45^, ^46^ ^]^ the solvation state of Mg^2+^ in solution significantly impacts reaction kinetics due to the fundamental similarities between alkali metals and alkaline earth metals. A recent comprehensive DFT modeling study on ethereal electrolyte solutions incorporating magnesium tetrakis(hexafluoroisopropoxyl)borate, Mg[B(HFIP)_4_]_2_, identified solvent‐dependent first Mg–O desolvation as the rate‐limiting step for Mg plating.^[^ ^47^ ^]^ The predicted trend in plating overpotential was in good agreement with the experimental observations, with the salt combined with diglyme (G2) exhibiting the lowest overpotential for Mg plating, regardless of the conductive salt used (**Figure** **2**).^[^ ^47^, ^48^, ^49^ ^]^ The desolvation energy landscape derived from DFT‐MD simulations further confirmed that [Mg(G2)_2_]^2^^+^ has a lower Mg–O desolvation energy than [Mg(G1)_3_]^2+^, supporting the notion that Mg plating is initiated by desolvation at the interface.^[^ ^47^ ^]^ This mechanistic understanding is also applicable to halide‐containing electrolyte solutions, where the relaxation of Mg^2+^–solvent interactions due to binding by negatively charged halide species facilitates the desolvation of solvating ethereal solvents at the interface. This phenomenon partially explains the superior plating/stripping kinetics observed in halide‐containing electrolytes.^[^ ^41^, ^50^ ^]^

As discussed above, the inherent stability of electrolyte components and the desolvation/dissociation barrier at the interface are the dominant factors determining Mg plating activity. Based on this fundamental understanding, well‐designed weakly coordinated anion (WCA)‐based ethereal solutions have been introduced as potential electrolytes for practical multivalent metal batteries.^[^ ^38^, ^48^, ^49^, ^51^, ^52^, ^53^, ^54^, ^55^ ^]^ These electrolytes exhibit excellent Mg plating/stripping performance, comparable to organoborate‐based electrolytes. However, their anodic stability is significantly improved due to the delocalization of negative charges on the anions, which enhances oxidation resistance. Despite these promising characteristics, the complex synthesis and high cost of WCA‐based electrolytes may hinder their large‐scale application in practical systems.

**Figure 3:**
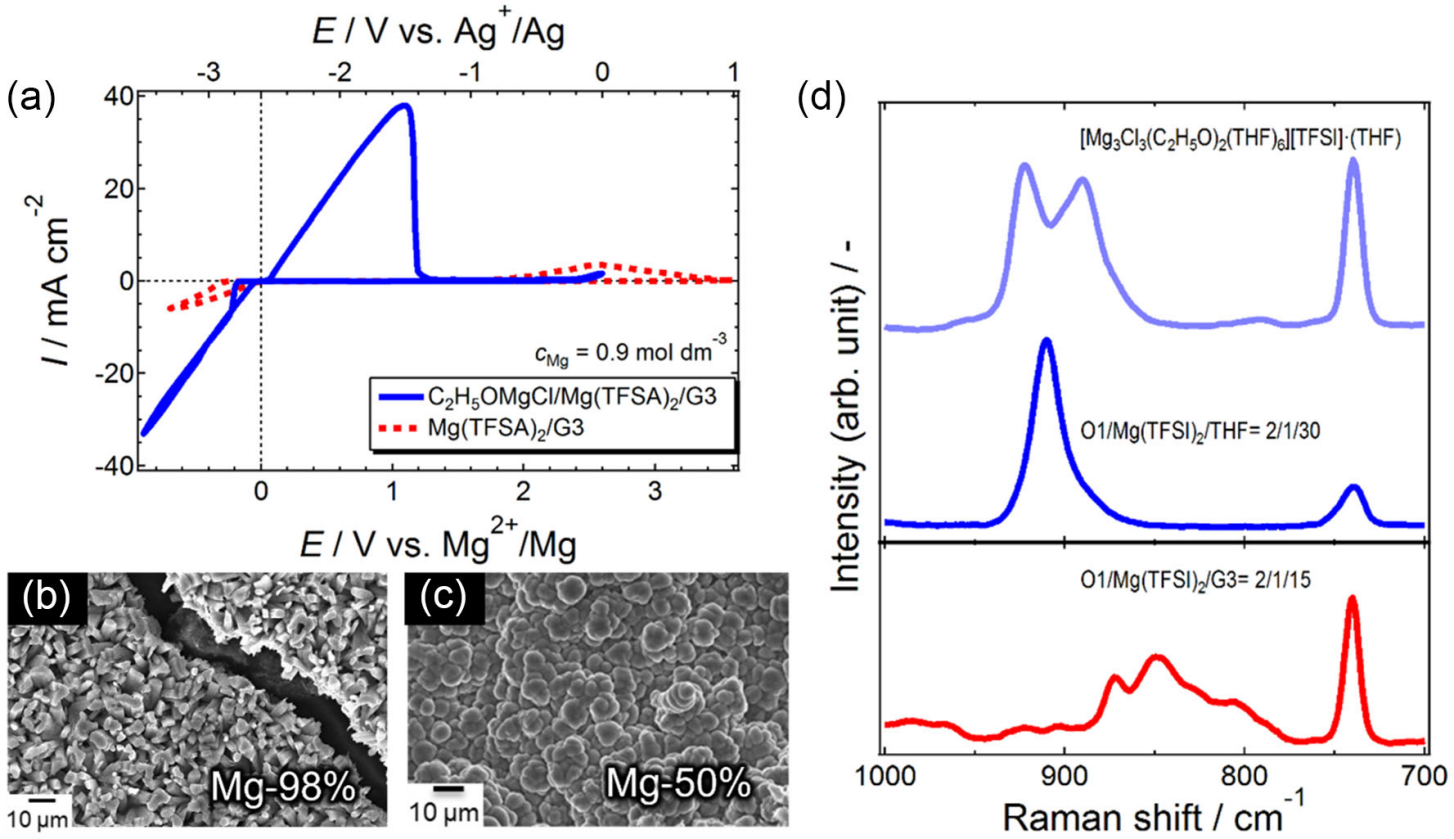
a) Cyclic voltammograms of two electrolyte systems containing (blue) dissociated TFSA anions and (red) associated TFSA anions. b,c) Scanning electron microscopy (SEM) images and Mg atomic ratio of deposits obtained from the respective systems. d) Corresponding Raman spectra. Reproduced with permission.^[^ ^62^ ^]^ Copyright 2017, The Royal Society of Chemistry.

Commercially available Mg(TFSI)_2_ and Mg(TfO)_2_ are potential candidates as conductive salts for RMMB electrolyte solutions. Ethereal solutions of Mg(TFSI)_2_ and Mg(TfO)_2_ exhibit somewhat reversible Mg plating/stripping activity.^[^ ^56^, ^57^, ^58^, ^59^ ^]^ However, systematic computational studies have revealed that the associative nature of these salts results in undercoordinated (bound) TFSI^−^, which is susceptible to reduction.^[^ ^15^, ^60^, ^61^ ^]^ The plating/stripping efficiency of these electrolyte solutions is closely linked to the coordination state of the anions in solution (**Figure** **3**). Regulating the solvation shell of Mg^2+^ through electrolyte formulation is a straightforward approach to optimizing electrolyte properties. Incorporating halide or BH_4_^−^ effectively modulates the solvation sheath due to the strong coordinating ability of these anions.^[^ ^35^, ^36^, ^59^, ^62^, ^63^, ^64^ ^]^ The resulting solutions, which contain well‐dissociated TFSI^−^ and TfO^−^, demonstrate superior efficiency compared to those with associated anions. However, this approach also introduces significant drawbacks, as halides and BH_4_^−^ exhibit corrosive behavior and poor anodic stability. An alternative strategy involves using strongly solvating solvents to dissociate associative anions while preserving the desirable characteristics of halide‐free electrolyte solutions.

### 2.2 Ether‐Nonether Dual Solvents and Hybrid System

Dual‐solvent (or cosolvent) approaches to enhance the electrochemical characteristics of ethereal Mg electrolyte solutions have been explored since at least 1933.^[^ ^21^ ^]^ Overcash et al. reported successful Mg electrolysis in a Grignard reagent (C_2_H_5_MgCl/ether) combined with a specific amine compound (dimethylaniline). Other organic solvents, including aniline, pyridine, acetate, sulfate, alcohol, and aryl compounds, were all unsatisfactory as cosolvents. The reactive nature of Grignard compounds toward carbonyl and alcohol compounds partly explains the unsuccessful electrolysis in these dual‐solvent systems. Later, an improved reaction efficiency for Mg plating/stripping cycling was reported by integrating triethylamine as a cosolvent in the (C_4_H_9_)_2_ Mg‐(C_2_H_5_AlCl_2_)_2_/THF system, known as the dichloro‐complex (DCC) solution.^[^ ^31^, ^65^ ^]^ By controlling the volume fraction of triethylamine relative to THF, the Mg cycling efficiency reached 99%.

Ionic liquids (ILs) are room‐temperature molten salts consisting solely of ions. Their unique physicochemical properties, including negligible volatility, unconventional solvation behavior, and high thermal, chemical, and electrochemical stability, make them promising electrolyte materials. However, typical single‐salt IL electrolyte solutions do not support reversible Mg plating/stripping,^[^ ^66^ ^]^ whereas high‐temperature molten salt systems composed of MgCl_2_ and additives are well known as baths for practical Mg electrolysis. Mg plating/stripping is feasible in acidic chloroaluminate baths containing MgCl_2_,^[^ ^67^ ^]^ again highlighting the critical role of Cl^−^ species in Mg electrochemistry. The addition of ILs to ethereal solutions has been found to improve ionic conductivity and enhance the reaction kinetics for Mg plating/stripping. Indeed, using ether‐IL dual solvents has been shown to increase current density and reduce overpotential for Mg deposition.^[^ ^68^ ^]^ Both the cations and anions of ILs significantly impact the electrochemical performance of the resulting electrolytes. A systematic study on CH_3_MgBr/THF‐IL dual‐solvent systems suggests that suppressing side reactions between reactive CH_3_MgBr and ILs through appropriate chemical modifications is essential for achieving electrolyte solutions with favorable electrochemical characteristics.^[^ ^69^, ^70^ ^]^ The structural flexibility of onium cations allows for functionalization of ILs. Imidazolium‐ and ammonium‐based ILs functionalized with oligoether groups can be regarded as hybrid systems. Introducing Mg(BH_4_)_2_ as a conductive salt into these ILs results in ether‐functionalized hybrid IL systems that exhibit (quasi‐)reversible Mg plating/stripping activity.^[^ ^71^, ^72^ ^]^

**Figure 4:**
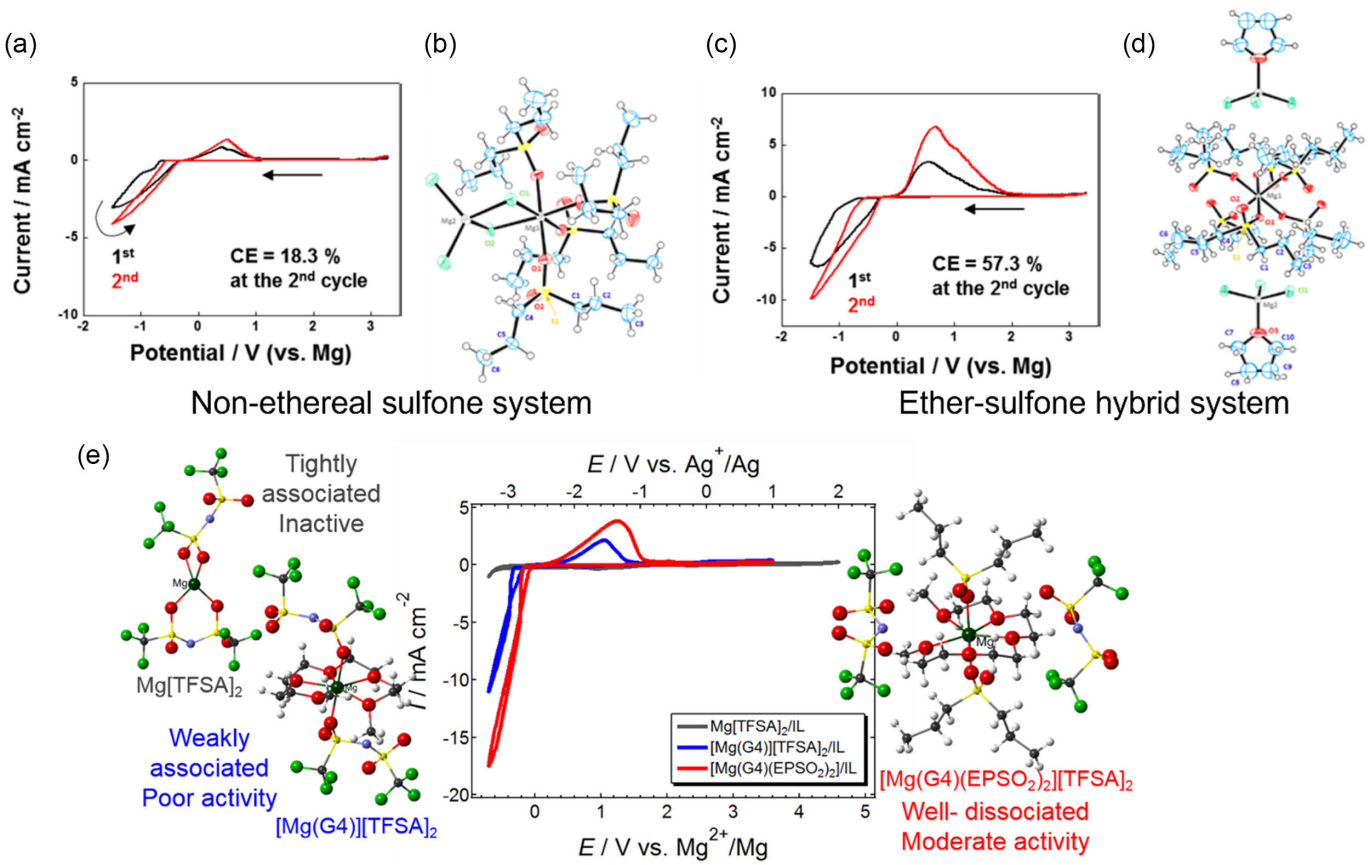
Cyclic voltammograms of Pt working electrodes recorded in a) 0.8 mol dm^−3^ MgCl_2_/DPSO_2_ and c) 0.8 mol dm^−3^ MgCl_2_/DPSO_2_‐THF (1/1, v/v) at 30 °C with a scan rate of 20 mV s^−1^; X‐ray crystal structures of possible active species in b) MgCl_2_/DPSO_2_ and d) MgCl_2_/DPSO_2_‐THF systems (Mg, gray; Cl, green; O, red; S, yellow; C, blue). Reproduced with permission.^[^ ^75^ ^]^ Copyright 2017, American Chemical Society. e) Relationship between the coordination state of TFSA in electrolytes and electrochemical Mg plating/stripping activity, where a well‐dissociated system enables improved plating/stripping performance. Reproduced with permission.^[^ ^15^ ^]^ Copyright 2019, The PCCP Owner Societies.

Inspired by the successful application of sulfone solvents in Al plating baths with AlCl_3_,^[^ ^73^ ^]^ sulfone‐based single‐ and dual‐solvent systems have been investigated as potential electrolyte solutions for RMMBs. While single‐salt electrolyte systems incorporating MgCl_2_ or Mg(TFSA)_2_ in sulfone solvents have demonstrated limited electrochemical activity for Mg plating/stripping,^[^ ^74^, ^75^ ^]^ combining them with ether has led to significantly improved performance.^[^ ^75^ ^]^ A similar enhancement in electrochemical characteristics has also been reported for certain ternary solvent systems incorporating ILs.^[^ ^15^ ^]^ A systematic structural study suggests that the coordination state of Mg^2+^ in MgCl_2_‐dipropyl sulfone (DPSO_2_) and Mg(TFSA)_2_‐dialkyl sulfone solutions changes in the presence of ether solvents, as shown in **Figure** **4**.^[^ ^15^, ^75^ ^]^ Overall, preventing counter anions from coordinating with Mg^2+^ to avoid the formation of transient, unstable species is crucial for developing electrolyte solutions with favorable electrochemical properties, as supported by computational studies.^[^ ^60^, ^61^ ^]^

**Figure 5:**
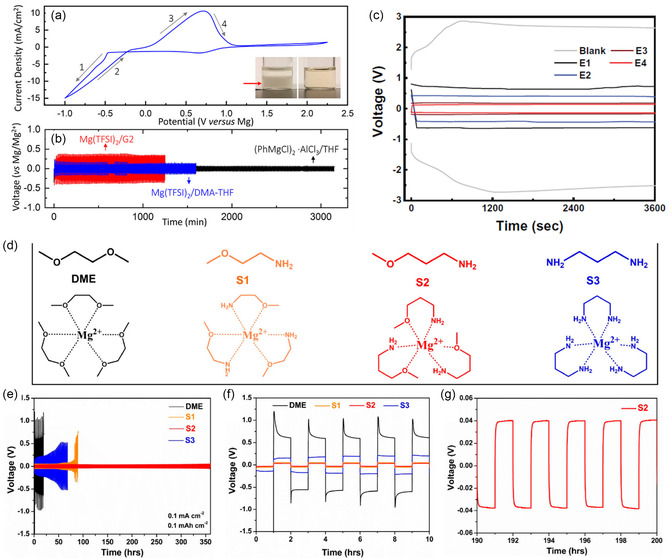
a) Cyclic voltammograms of 0.5 mol dm^−3^ Mg(TFSA)_2_ in 2.0 mol dm^−3^ DMA‐THF at a scan rate of 50 mV s^−1^; b) galvanostatic cycling of symmetric Mg/Mg cells using 0.5 mol dm^−3^ Mg(TFSA)_2_ in 2.0 mol dm^−3^ DMA‐THF (blue), 0.5 mol dm^−3^ APC (black), and 0.5 mol dm^−3^ Mg(TFSA)_2_ in G2 (red) electrolytes at a current density of 20 μA cm^−2^. Reproduced with permission.^[^ ^23^ ^]^ Copyright 2020, American Chemical Society. c) Overpotentials at the 10th plating/stripping cycle in Mg||Mg cells at 1.5 for 1.5  m^−2^, recorded in the blank (G1) and G1‐alkoxyamine dual solvent systems. Reproduced with permission.^[^ ^24^ ^]^ Copyright 2021, AAAS. d) Structures of solvent molecules and their speculated solvated structures with Mg^2+^ ions; e–g) galvanostatic cycling performance of G1, S1, S2, and S3 solvents with 0.1 mol dm^−3^ Mg(TFSA)_2_ in Mg//Mg symmetric cells at 0.1 for 0.1 mAh cm^−2^. Reproduced with permission.^[^ ^26^ ^]^ Copyright 2023, American Chemical Society.

Due to the strong donating ability of amines, their integration as cosolvents effectively regulates the solvation sheath of Mg^2+^ in ethereal solutions. While amines have previously been introduced in Grignard‐based and organohaloaluminate solutions,^[^ ^21^, ^65^ ^]^ their application to simple ethereal solutions of Mg(TFSA)_2_ salt was first reported in 2020.^[^ ^23^ ^]^ The addition of dimethylamine (DMA) significantly enhances the solubility of Mg(TFSA)_2_ in ethereal solutions and improves electrochemical Mg plating/stripping performance, attributed to the strong solvation ability of DMA (**Figure** **5**a,b). Interestingly, single crystals grown from Mg(TFSA)_2_/DMA‐THF solutions were identified as Mg(TFSA)_2_(THF)_4_ adducts, where DMA does not participate in Mg^2+^ coordination, likely due to the thermodynamic instability of DMA‐coordinated Mg^2+^ complexes.

Building on this, a groundbreaking concept of solvation sheath reorganization using amine cosolvents was introduced in 2021 (Figure 5c).^[^ ^24^ ^]^ Ether‐amine dual solvent systems enable remarkable Mg plating/stripping performance while reducing parasitic reactions by modulating the solvation sheath, thereby minimizing desolvation overpotential at the positive electrode–electrolyte interface. Following this pioneering work, highly efficient dual and hybrid electrolyte solutions have been reported. Yang et al. and Wang et al. independently explored the correlation between coordination chemistry and electrochemical performance in a series of alkylalkoxyamine‐based systems (Figure 5d–g), concluding that the charge transfer kinetics and desolvation process are in competition, primarily governed by the solvation ability of the solvent molecules.^[^ ^25^, ^26^ ^]^ The latter study also demonstrated a remarkable water‐resistant property in specific systems.

Beyond Mg(TFSA)_2_‐based systems, the same approach has been applied to Mg(TfO)_2_‐based electrolytes, where a 2‐methoxyethylamine (MOEA)‐G2 dual solvent system exhibited significantly improved physicochemical and electrochemical properties.^[^ ^27^ ^]^ The preferred decomposition of MOEA‐coordinated Mg‐ion complex accompanied by (TfO)^−^ decomposition would result in the gradient organic–inorganic interface, which enables a fast interfacial kinetics and uniform Mg plating.^[^ ^76^ ^]^ The comprehensive mechanistic work by Li et al. demonstrated that H_2_ evolution from amine group reduction was the main parasitic reaction and mitigation of such unfavorable reaction by combining the rationally designed less‐coordinating amine solvent (methoxypropylamine; MOPA) and associative (TfO)^−^ anion can improve the stability and reversibility of Mg plating.^[^ ^77^ ^]^ Notably, according to the present classification, solutions containing alkylalkoxyamines are categorized as (intramolecular) ether–nonether hybrid systems rather than purely nonether systems, as alkylalkoxyamines contain a C—O—C unit within their molecular structure.

Another dual‐solvent system proposed for RMMB electrolytes consists of ether and trialkylphosphate. Zhao et al. introduced trimethyl phosphate (TMP) as a competing agent against G1 for Mg^2+^ solvation in Mg(TFSA)_2_/G1 solutions.^[^ ^78^ ^]^ The tailored solvation sheath, assisted by the favorable interlayer formation upon organophosphorus decomposition, enhances charge transport kinetics. The concept of phosphate integration was further explored by Wang et al. who demonstrated that the addition of LiTfO to Mg(TFSA)_2_‐TMP‐G1 systems effectively promoted electrochemical reaction kinetics and cycling stability, benefiting from high‐entropy electrolyte formulations.^[^ ^79^ ^]^ Outstanding performance was achieved using Mg(TfO)_2_ as a conductive salt and a G2‐triethyl phosphate (TEP) dual solvent system.^[^ ^80^ ^]^ The well‐designed, intrinsically stable electrolyte enabled Mg deposition into densely aligned hexagonal platelets due to minimal electrolyte decomposition during plating/stripping. The optimized electrolyte formulation exhibited exceptional plating/stripping efficiency and an extended cycle life, even at practical areal capacities. These strategies, which weaken ion pairing and regulate solvation sheath through solvent modulation, open new frontiers for designing rational electrolyte solutions to realize high‐power and high‐energy‐density RMMBs.

## 3 Mg Plating in Nonethereal Solutions

### 3.1 Single Nonether System

Achieving Mg plating in nonaqueous, nonethereal media would overcome several critical obstacles in realizing RMMBs; however, this remains a long‐standing challenge due to the highly selective compatibility of Mg metal with common electrolyte components. An anodic response attributable to Mg stripping has been observed in various electrolyte systems, including acetonitrile (AN), propylene carbonate (PC), diethyl carbonate (DEC), γ‐butyrolactone (GBL), dimethylformamide (DMF), dimethyl sulfoxide (DMSO), dimethyl sulfite (DMS), and conventional IL solvents with Mg salts.^[^ ^9^, ^17^, ^21^, ^81^, ^82^, ^83^ ^]^ Microscopic analysis of Mg electrodes has provided evidence of Mg stripping after both static and dynamic polarization in these nonethereal systems. However, none of them support measurable Mg plating, despite the presence of cathodic responses in cyclic voltammograms. Special attention must be given to the possibility of solvent reduction at the desired electrode potential, as demonstrated in the Mg(TFSA)_2_/AN system, which undergoes reductive decomposition at ≈−0.2 V vs. Mg without any observable Mg plating.^[^ ^81^ ^]^ It has been suggested that transient Mg^0^ is too reactive in these solvents and undergoes side reactions, preventing plating.^[^ ^9^, ^81^, ^82^ ^]^ Later computational studies, supported by advanced spectroscopic techniques, confirmed that transient Mg^+^‐solvent and Mg^2+^‐solvent complexes bypass reduction and/or decompose instead of forming metallic Mg, ultimately leading to the formation of an insulating passivation layer on the Mg surface.^[^ ^60^, ^61^, ^84^ ^]^

A pioneering study on an electrochemically active nonethereal Mg electrolyte was reported by Senoh et al. in 2014.^[^ ^74^ ^]^ Reversible Mg plating/stripping was observed in electrolyte systems composed of specific linear and cyclic sulfone solvents combined with Mg(TFSA)_2_. Morphological and diffraction analyses of deposits obtained by potentiostatic electrolysis confirmed successful Mg metal plating, though with poor current efficiency, high overpotential, and low deposit purity. Subsequent studies using Mg[B(HFIP)_4_]_2_ in sulfone‐based electrolytes also indicated Mg plating, but anodic stripping currents remained negligible.^[^ ^17^ ^]^ This suggests that solvent decomposition during the cathodic process hinders efficient Mg stripping. Improved electrochemical performance was achieved by replacing Mg(TFSA)_2_ with MgCl_2_, increasing the plating/stripping efficiency on Pt electrodes from 5% in Mg(TFSA)_2_/SL to 18.3% in MgCl_2_/DPSO_2_ systems (Figure 4),^[^ ^75^ ^]^ highlighting the critical role of Cl^−^ species in Mg plating/stripping reactions.

**Figure 6:**
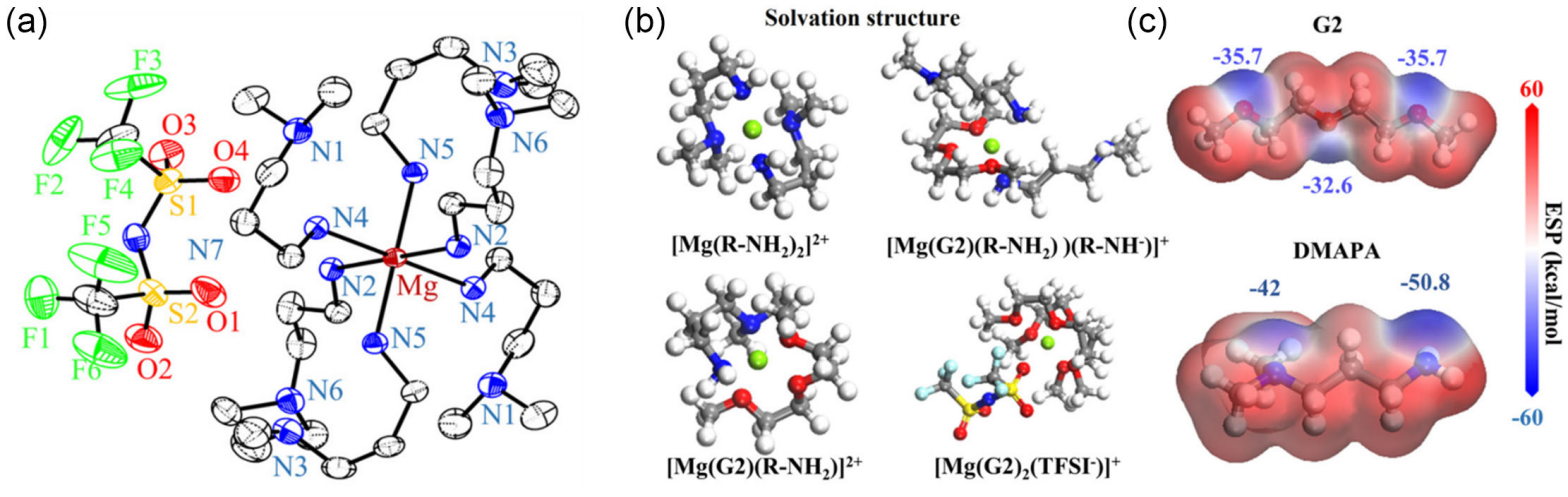
a) The ORTEP diagram of the single‐crystal [Mg(DMAPA)_6_](TFSA)_2_ structure (50% thermal probability ellipsoid). The hydrogen atoms and the disordered second components are omitted for clarity. b) Simulated configurations of electrolyte species. c) The ESP distribution in G2 and DMAPA solvents. Reproduced with permission.^[^ ^28^ ^]^ Copyright 2024, The Royal Society of Chemistry.

Another nonethereal electrolyte system exhibiting Mg plating/stripping activity utilizes alkyldiamines as solvents. Reversible Mg plating/stripping with relatively high Coulombic efficiency has been demonstrated in systems containing ethylenediamine, 1,3‐diaminopropane, *N*,*N*’‐dimethylethylenediamine, and 3‐dimethylaminopropylamine combined with Mg(TFSA)_2_ and Mg[B(HFIP)_4_]_2_ salts (Figure 5d–f).^[^ ^24^, ^26^ ^]^ X‐Ray crystallography and spectroscopic analysis, alongside electrochemical characterization, suggest that the [Mg(amine)_6_]^2+^ complex is the active ionic species in these media (**Figure** **6**).^[^ ^28^ ^]^ Additionally, partially protonated alkylamines facilitate the formation of detectable MgH_2_ components in the deposits, which may mitigate electrolyte decomposition on Mg electrodes and enhance plating/stripping kinetics. The positive role of MgH_2_‐containing SEI originated from parasitic interfacial reaction between Mg metal and alkylamines has also been pointed out in the different work, where the ether‐amine dual solvent system was employed.^[^ ^85^ ^]^ The critical role of reactive protons in alkylamines was further supported experimentally, as the *N*,*N*,*N*’,*N*’‐tetramethylethylenediamine‐based electrolyte—lacking active protons—exhibited inferior Mg plating/stripping characteristics.^[^ ^24^ ^]^

Despite their promising performance, a significant drawback of alkylamines as electrolyte solvents for RMMBs is their poor anodic stability compared to conventional ether solvents.^[^ ^25^ ^]^ Oxidative decomposition of amines occurs at relatively lower potentials, which poses a challenge for achieving high‐energy‐density RMMBs.

Considering the above experimental findings, it is evident that significant barriers remain for achieving Mg plating in nonethereal solution systems. Electrolyte solvents for RMMB applications must exhibit a balanced combination of properties to support both bulk transport and interfacial electrochemical reactions. Given the complexity of these processes, it is challenging to achieve multiple functions within a single solvent molecule. For instance, incorporating anti‐oxidation groups into the molecular structure may compromise the stability of the solvent against reduction.^[^ ^17^ ^]^ To address these interrelated challenges, a strategic approach involving the division of solvent functions among multiple components should be pursued.

### 3.2 Mg Electrochemistry in Nonether System with an Aid of Artificial Interface

Constructing artificial interfaces on Mg electrodes is a promising strategy for enabling high‐performance RMMBs with nonether‐based electrolyte systems. By preventing direct contact between the electrolyte and the highly reductive Mg surface through an Mg^2+^‐conductive artificial interface, electrolyte systems with lower compatibility against bare Mg can be utilized. While this approach has the potential to expand the selection of viable electrolyte components, most attempts using bare Mg foils have been unsuccessful, likely due to the lack of uniformity in the artificial interface. The surface of Mg foils is typically covered with an uneven natural passivation layer composed mainly of magnesium oxide, hydroxide, and carbonate, which leads to nonuniform artificial interphase formation.^[^ ^86^ ^]^

**Figure 7:**
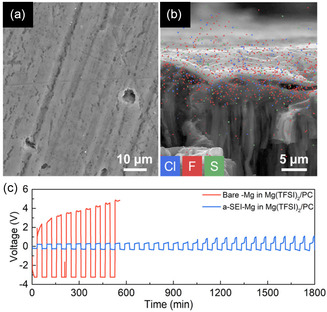
SEM images of a) the surface and b) the cross section with EDS mapping of an Mg foil soaked in AlCl_3_ + LiTFSA/TEGDME for 24 h. c) Voltage responses of bare‐Mg/bare‐Mg and a‐SEI‐Mg/a‐SEI‐Mg cells in an Mg(TFSA)_2_/PC system at a current density of 0.01 mA cm^−2^. Reproduced with permission.^[^ ^20^ ^]^ Copyright 2021, American Chemical Society.

To address this issue, Son et al. developed an innovative method to construct a highly uniform Mg^2+^‐conductive artificial interface using Mg powder instead of bulk Mg foil.^[^ ^19^ ^]^ By coating Mg powder with a polyacrylonitrile (PAN) solution containing Mg(TfO)_2_ and subsequently inducing thermal cyclization, they achieved a uniform Mg(TfO)_2_‐integrated PAN composite. In this system, Mg(TfO)_2_ facilitates Mg^2+^ conduction, while the low electronic conductivity of the artificial interphase prevents undesirable electrolyte reduction, enabling the use of carbonate solvents in RMMB operation. This approach allowed a highly reversible Mg plating/stripping process in the Mg(TFSA)_2_/PC system. Similarly, Mg foil electrodes treated with Li(TFSA)‐AlCl_3_‐G4 demonstrated Mg plating/stripping activity in the same electrolyte (**Figure** **7**).^[^ ^20^ ^]^ The treatment involved etching the native passivation layer with strongly Lewis acidic AlCl_3_, followed by the formation of an Mg^2^^+^‐conductive, electronically insulating interphase through reactions with Cl, F, and S species.

Various Mg^2+^‐conductive artificial interphases have been reported, yet their application in nonethereal electrolyte systems remains limited. Further studies are necessary to elucidate the fundamental mechanisms governing interfacial Mg plating/stripping in nonethereal electrolytes, paving the way for more effective artificial interface designs.

## 4 A Protocol to Judge Mg Plating in Nonethereal Solutions

The number of studies on dual, hybrid, and nonether solvent systems has increased in recent years. The regulation of the solvation sheath through electrolyte formulation adjustments is a feasible approach that is readily accessible to researchers who may not have a background in organic chemistry. This accessibility has stimulated research efforts toward developing practical electrolytes for RMMB applications. However, as noted earlier, some studies have proposed novel but unconventional electrolyte formulations with limited experimental evidence supporting Mg plating activity.

**Figure 8:**
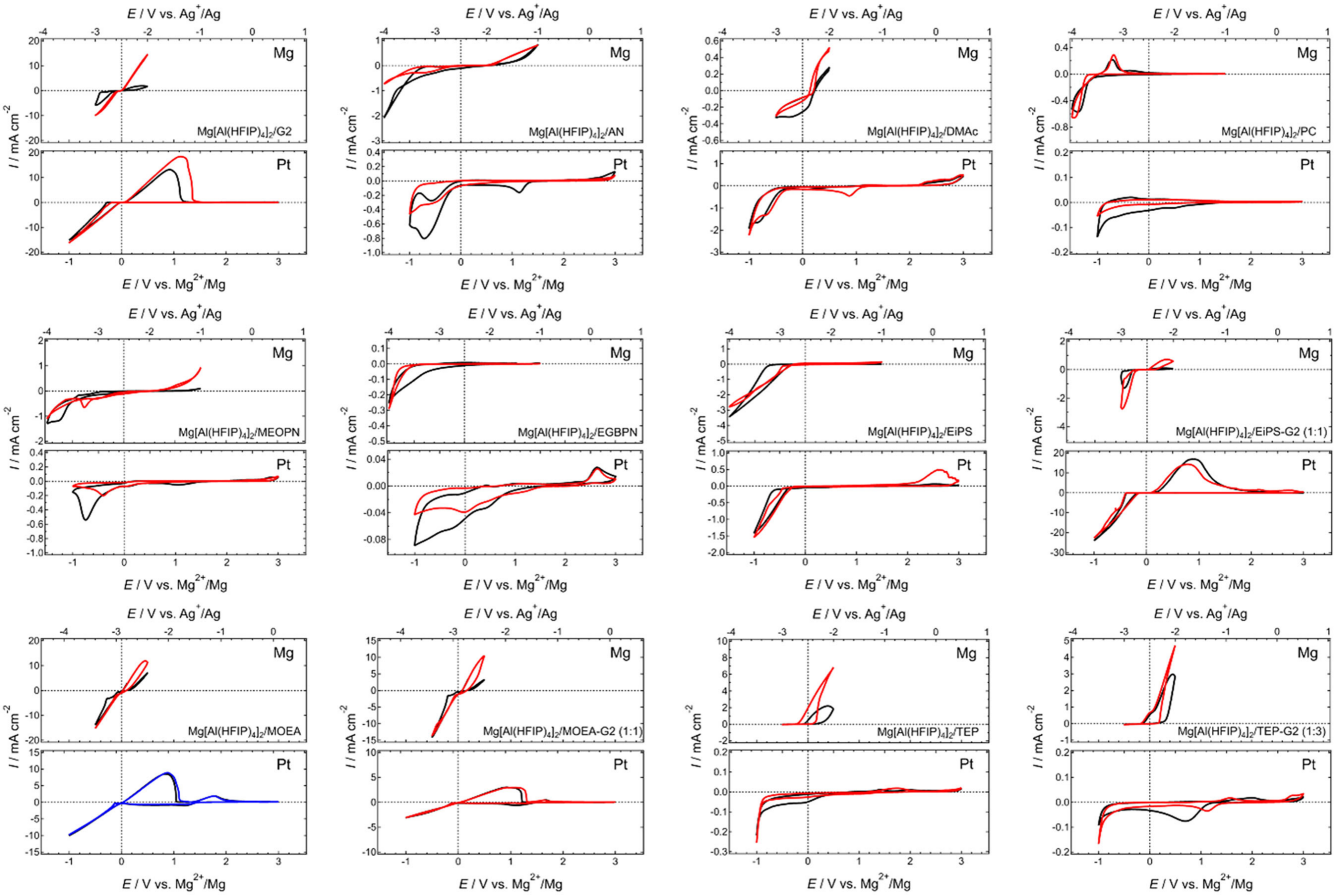
Cyclic voltammograms of (upper) Mg and (lower) Pt working electrodes recorded in a series of 0.3 mol dm^−3^ Mg[Al(HFIP)_4_]_2_‐based electrolyte solutions at a scan rate of 10 mV s^−1^ at 30 °C. Black, blue, and red curves represent the 1st, 5th, and 10th cycles, respectively. All voltammograms shown here are originally created for this review.

**Figure 9:**
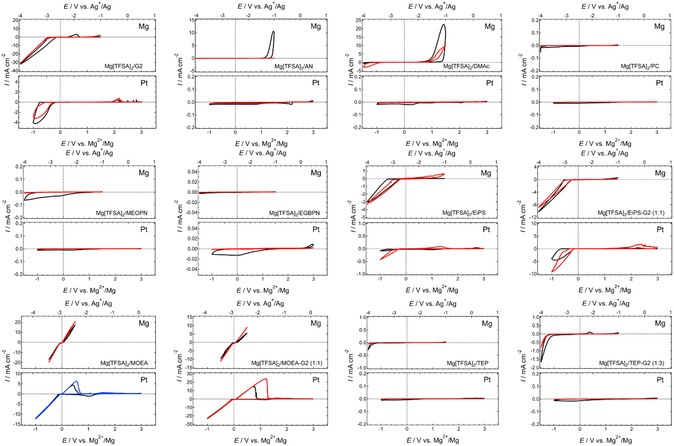
Cyclic voltammograms of (upper) Mg and (lower) Pt working electrodes recorded in a series of 0.5 mol dm^−3^ Mg[TFSA]_2_‐based electrolyte solutions at a scan rate of 10 mV s^−1^ at 30 °C. Black, blue and red curves represent the 1^st^, 5^th^, and 10^th^ cycles, respectively. All voltammograms shown here are originally created for this review.

A comprehensive solvent survey using Mg[B(HFIP)_4_]_2_, in which Mg^2+^ and [B(HFIP)_4_] are well dissociated due to the weak coordination ability of the anion, indicated poor compatibility of nitriles and acid ester compounds—including carbonate, amide, phosphate, and borate—with Mg plating.^[^ ^17^ ^]^ To assess the universality of this observation, this study extends the survey to systems incorporating other representative conductive salts, Mg(TFSA)_2_ and Mg[Al(HFIP)_4_]_2_. The cyclic voltammograms of various single salt‐solvent systems are summarized in **Figure** **8** and **9**. Additionally, dual solvent systems such as ether‐alkoxyamine, ether‐sulfone, and ether‐phosphate—each previously reported to exhibit favorable Mg plating/stripping activity in the literature^[^ ^24^, ^28^, ^75^, ^78^, ^80^ ^]^—are included. Mg[Al(HFIP)_4_]_2_ isolated as an adduct in which three G1 molecules coordinate to Mg^2+^ was synthesized according to the literature.^[^ ^48^ ^]^ Given the strong influence of working electrodes on electrochemical reactions, measurements were conducted with Pt and Mg working electrodes in a three‐electrode setup, using Mg ribbon as the counter electrode and Ag^+^/Ag with a liquid junction as the reference electrode. The surface and solution were also visually inspected after cycling to ensure that the observed reductive current response was correctly attributed to Mg plating. This systematic screening, combined with post‐experimental observations, further confirms that only a limited range of solvents enable electrochemical Mg plating.

As reported previously, no response indicative of reversible Mg plating/stripping has been observed in systems incorporating conventional solvents such as nitriles, amides, and carbonates. Ether‐nitrile hybrid systems (ethylene glycol bis(propionitrile) ether, EGBPN; 3‐methoxypropionitrile, MeOPN) also fail to support the desired electrochemical reactions, highlighting the extremely poor compatibility of nitriles with Mg. Somewhat reversible responses observed for Mg working electrodes in dimethyl acetamide (DMAc), TEP, and PC‐based systems mimic Mg plating; however, the exact nature of these responses remains unclear. Although excellent Mg plating/stripping performance has been reported for ether‐phosphate dual solvent systems,^[^ ^78^, ^80^ ^]^ the integration of G2 in this study does not significantly impact electrochemical performance, regardless of the conductive salt used. The discrepancy in results may be attributed to impurities or other unidentified factors.

In contrast, methoxyethylamine (MOEA)‐based systems exhibit remarkably reversible responses. Visual inspection of the working electrodes confirms the presence of Mg deposits, indicating that the observed electrochemical responses correspond to Mg plating/stripping reactions. Performance, particularly plating/stripping efficiency, further improves in ether‐MOEA dual solvent systems, aligning well with previous studies.^[^ ^24^, ^27^ ^]^ Dialkylsulfone‐based systems show intermediate behavior; while single ethyl isopropyl sulfone (EiPS) solvent systems exhibit poor electrochemical performance, EiPS‐G2 dual solvent systems demonstrate noticeable improvement. This suggests that Mg plating in such media may involve the reductive decomposition of dialkylsulfones, as these solvents display moderate compatibility with Mg plating/stripping reactions.

**Figure 10:**
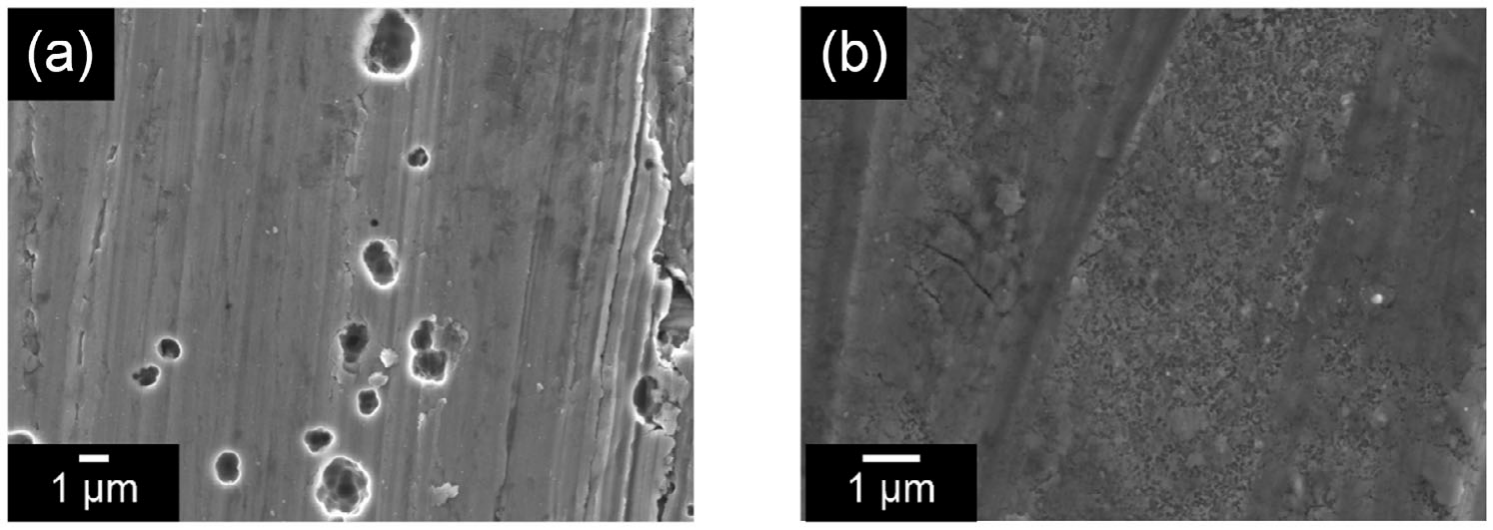
SEM images of Mg electrodes cycled in 0.3 mol dm^−3^ a) Mg[Al(HFIP)_4_]_2_/DMAc and b) Mg[Al(HFIP)_4_]_2_/TEP. No deposit assignable to Mg plating is discernible. The images are originally acquired for this review.

SEM analysis of cycled Mg electrodes in nitrile, amide, carbonate, and phosphate‐based nonethereal systems indicates Mg dissolution in these media. However, no clear evidence of Mg plating is discernible in the SEM images (**Figure** **10**). The somewhat reversible cathodic and anodic responses observed in electrochemical profiles likely stem from oxidative electrolyte decomposition associated with Mg dissolution (i.e., corrosion) and subsequent reductive decomposition of species formed during the anodic process. The negligible response of Pt working electrodes in these media further supports this interpretation. Computational studies on electron affinity reinforce the poor compatibility of these nonethereal solvents with Mg plating, as their electron affinities are significantly higher than those of ethers.^[^ ^17^ ^]^ These results serve as a cautionary note: reversible current responses on Mg electrodes do not necessarily indicate active Mg plating/stripping. This issue becomes even more complex when Mg electrodes are modified with artificial interfaces.

According to these findings, researchers must exercise particular caution when interpreting results from newly developed RMMB electrolyte materials, especially those based on nonethereal solvents. A galvanostatic cycling study using symmetric Mg–Mg electrode setups is insufficient for evaluating electrolyte performance, as the observed currents do not necessarily correspond to Mg plating/stripping reactions. Additionally, the symmetric cell configuration may cause undetectable shifts in equilibrium potential due to side reactions between the electrolyte and Mg electrodes, leading to misinterpretation of observed responses as Mg plating. Moreover, subsequent morphological, structural, and chemical analyses of cycled Mg electrodes may not provide definitive evidence of electrochemical activity.

**Scheme 2:**
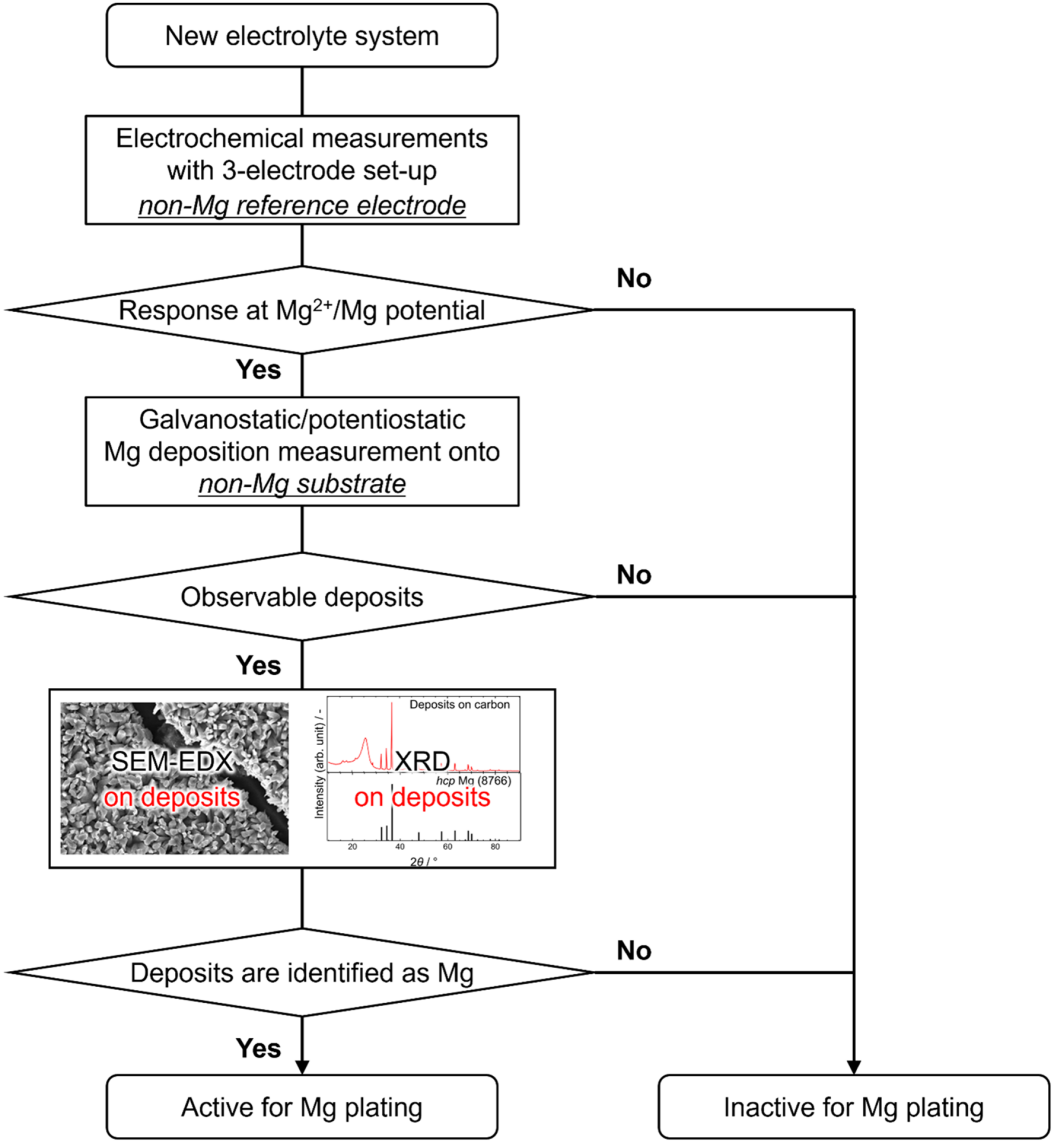
Proposed validation protocol (flowchart) of Mg plating activity for new electrolyte systems.

To validate Mg plating capability, a systematic approach is recommended: 1) conduct cyclic voltammetry or chronoamperometry/chronopotentiometry using a three‐electrode setup with a reliable reference electrode; 2) deposit Mg onto Mg‐free substrates under galvanostatic or potentiostatic conditions; and 3) characterize the deposits using SEM and X‐ray diffraction (XRD) (**Scheme** **2**). XRD is particularly valuable, as Mg metal exhibits distinct diffraction patterns that confirm electrochemical deposition.

**Figure 11:**
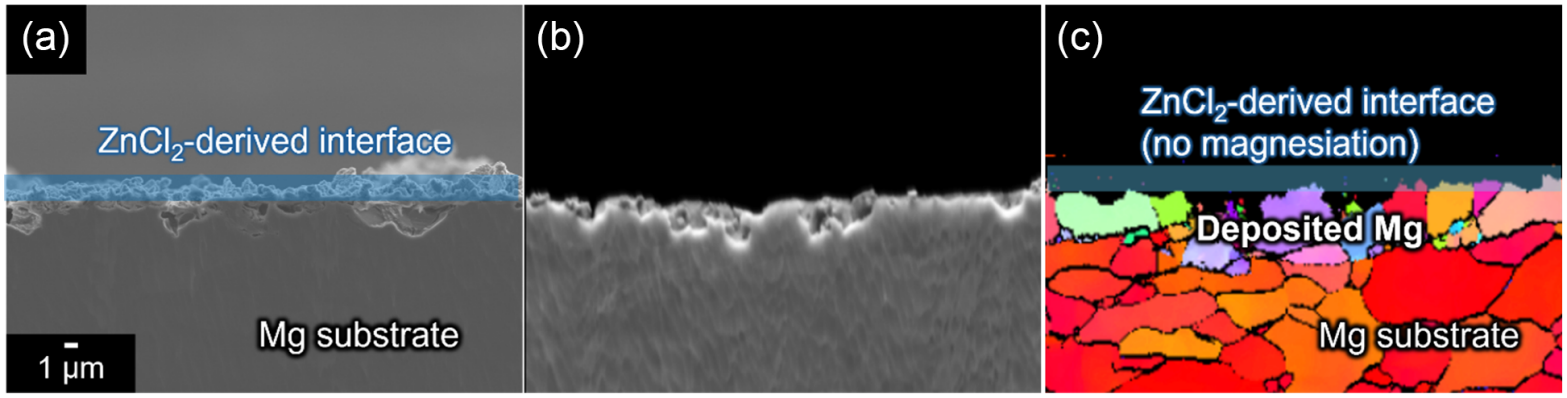
a) Cross‐sectional SEM images and corresponding b) forward‐scattered electron detection diode image and c) inverse pole figure map of ZnCl_2_‐modified Mg electrodes after cycling. The figures are originally created for this review while the same experimental conditions as ref.^[^ ^86^ ^]^ were adopted.

For Mg electrodes modified with artificial interfaces, more advanced analyses are necessary. Electron backscatter diffraction (EBSD) is a powerful technique for verifying Mg plating beneath artificial interface,^[^ ^86^ ^]^ as the grain orientation and size of deposited Mg should differ from those of the parent Mg substrate. EBSD analysis of the cross section provides direct evidence of Mg deposition beneath the interface (**Figure** **11**). High‐angle annular dark‐field scanning transmission electron microscopy (HAADF‐STEM) is also effective for identifying the boundary where Mg plating occurs.^[^ ^87^ ^]^ While these methods require specialized analytical equipment that may not be readily accessible to all researchers, adopting proper validation techniques is crucial to ensuring accurate assessments and preventing misconceptions that could mislead future studies.

## 5 Concluding Remarks

**Figure 12:**
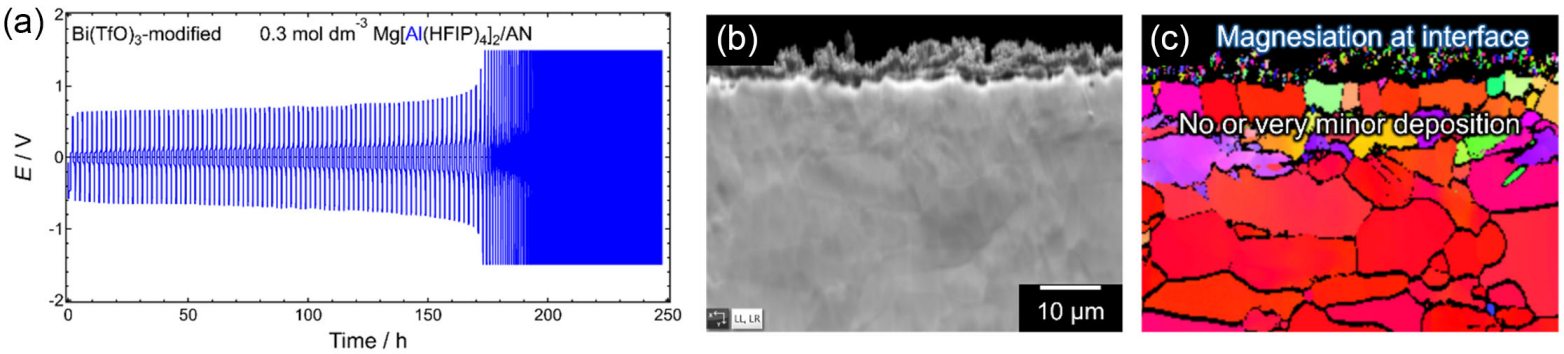
a) Galvanostatic cycling profiles of a symmetric cell with Bi(TfO)_3_‐modified Mg electrodes measured in 0.3 mol dm^−3^ Mg[Al(HFIP)_4_]_2_/AN at a current density of 0.1 mA cm^−2^. b) Forward scattered electron detection diode image and c) inverse pole figure map of the cross‐sectional Mg electrodes after cycling, obtained via EBSD analysis. The figures are originally created for this review.

An overview of Mg electrochemistry in ethereal and nonethereal electrolyte systems has been presented in this article, along with a proposed protocol for determining whether the proposed (uncommon) systems are active for Mg plating/stripping. Comprehensive studies on electrochemical Mg plating/stripping activity across various electrolyte systems reaffirm that only a limited range of solvents, including ethers, amines, and dialkylsulfones, support reversible Mg plating/stripping. The common working principle that governs the activity can be the intrinsic chemical stability of the solvents against reductive Mg metal. Upon regulating solvation environment through compositional modification, the parasitic reactions can be mitigated and favorable electrochemical performance can be achieved in these media. In contrast, Mg plating is largely inhibited in acid ester‐based systems, such as carbonate, acetate, and phosphate, due to severe side reactions with electrolyte components. Systematic cyclic voltammetry studies using a three‐electrode setup further highlight the risk of misinterpretation, as seemingly reversible anodic/cathodic current responses with Mg electrodes can sometimes be misleading. To prevent such misjudgments, researchers must carefully analyze their results and provide solid evidence of Mg plating using appropriate analytical techniques. SEM observations, elemental analysis, and XRD diffraction of Mg deposits on Mg‐free substrates should be presented as proof of Mg plating. In cases where Mg electrodes with artificial interfaces are used, more advanced analytical techniques such as EBSD and HAADF‐STEM are necessary to confirm Mg plating activity in nonethereal systems. EBSD is particularly suitable for this purpose, as it enables visualization of grain orientation differences and strain at grain boundaries over a relatively large area compared with STEM analysis. EBSD profiles obtained after galvanostatic cycling in the Mg[B(HFIP)_4_]_2_/AN system with Bi(TfO)_3_‐modified Mg electrodes clearly revealed that the observed profiles corresponded mainly to magnesiation/demagnesiation of the Bi‐based interlayer rather than Mg plating/stripping (**Figure** **12**), as no Mg deposits with different orientations were detected on the Mg substrate. This finding further underscores the importance of rigorous analysis in studies of Mg electrochemistry in uncommon nonethereal systems to prevent false conclusions.

The characteristics of the representative ethereal and nonethereal solutions is tabulated in **Table** **1**. The electrochemical performance of the nonethereal electrolyte systems is apparently inferior to that of the ethereal systems. It should be noted here that the electrochemical properties are strongly affected by various experimental conditions including applied current density, areal capacity, cut‐off voltage, counter electrode, separator material, measurement temperature, sweep rate, sweep range, and cell configuration. Therefore, a fair comparison among different works is, in principle, impossible and a summary table of these properties is often misleading. Researchers must exercise particular caution again when comparing the performances from different literatures. Anyhow, the electrochemical performance of the RMMB electrolytes has greatly been improved especially in recent years. However, the compatibility of the present ethereal solutions including single, hybrid, and dual solvent systems against high‐voltage positive electrodes is still unsatisfactory.^[^ ^88^, ^89^ ^]^ Development of anodically stable electrolyte systems with Mg plating/stripping capability remains a long‐standing challenge in the RMMB field. Moving away from ethereal solutions is a direct approach to mitigating the issues associated with ethereal solvents; however, Mg plating rarely occurs in nonethereal polar solvent‐based systems due to the highly reactive nature of Mg metal and transient Mg species. Making Mg‐compatible solvents anodically stable is an alternative approach to overcome the above issues. Introduction of electron‐withdrawing fluorine and fluoroalkyl groups onto the ether structure was unfortunately unsuccessful due to the associated collective shift of cathodic stability.^[^ ^17^ ^]^ A unique interfacial characteristics of amine solvents opens new approach in designing rational electrolyte systems by adopting appropriate chemical modification on the molecular structure and/or application of functional additives. An ideal cathode electrolyte interface would avoid undesired interfacial reactions and facilitate favorable interfacial charge transfer reactions.^[^ ^90^ ^]^ Anyhow, it is crucial for the RMMB research community to establish clear criteria for determining whether proposed systems genuinely support electrochemical Mg plating, ensuring that conclusions are not drawn based on uncertain experimental data.

**Table 1:** Summary of electrochemical properties of representative ethereal and nonethereal electrolyte systems.

Conductive salt(s) Mg species	Main solvent	Cosolvent [vol%]	Mg plaiting activity	Polarization/V	Cycle life	Coulombic efficiency/%	Anodic stability (substrate)/V vs. Mg	References
*Simple ethereal electrolytes*								
MgBr_2_, Mg(SCN)_2_, Mg(ClO_4_)_2_, Mg(OC_2_H_5_)_2_, Mg(OCH_3_)_2_	(C_2_H_5_)_2_O		No					[21]
*x*(*n*‐C_4_H_9_)_2_ Mg‐*y*(C_2_H_5_)AlCl_2_	THF							[65]
*x* = 1, *y* = 2			Yes	–	–	95 (CV)a)	2.10 (Pt)	
*x* = 1, *y* = 1			Yes	–	–	96 (CV)a)	2.05 (Pt)	
(C_6_H_5_)_2_ Mg‐2(C_2_H_5_)AlCl_2_	THF		Yes	–	–	80 (CV)a)	2.08 (Pt)	
*x*(*n*‐C_4_H_9_)_2_ Mg‐*y*AlCl_3_	THF							
*x* = 1, *y* = 2			yes	–	–	75 (CV)a)	2.40 (Pt)	
*x* = 1, *y* = 1			Yes	–	–	86 (CV)a)	2.10 (Pt)	
(*n*‐C_4_H_9_)_2_ Mg‐B(C_6_H_5_)_3_	THF		Yes	–	–	68 (CV)a)	1.60 (Pt)	
(*n*‐C_4_H_9_)_2_ Mg‐BCl_3_	THF		Yes	–	–	80 (CV)a)	1.20 (Pt)	
(*n*‐C_4_H_9_)_2_ Mg‐2(C_2_H_5_)AlCl_2_	THF		Yes	0.25	–	100 (CV)a)	–	[91]
2C_6_H_5_MgCl‐AlCl_3_	THF		Yes	0.20	–	100 (CV)a)	>3 (Pt)	
Mg(TFSA)_2_	G2		Yes	1.99 @0.1 mA cm^−2^	<30 @0.1 mA cm^−2^, 0.1 mAh cm^−2^	40 @0.1 mA cm^−2^, 0.1 mAh cm^−2^	–	[28]
Mg(TFSA)_2_‐MgCl_2_	G1	THF (75)	Yes	0.25 @1 mA cm^−2^	>700 @1 mA cm^−2^, 1 mAh cm^−2^	98.8 @1 mA cm^−2^, 1 mAh cm^−2^	–	[92]
Mg(TfO)_2_‐MgCl_2_	G1		Yes	0.34 @0.5 mA cm^−2^	>100 @0.5 mA cm^−2^, 0.5 mAh cm^−2^	99.1 @0.5 mA cm^−2^, 0.5 mAh cm^−2^	3.0 (Pt)	[59]
Mg(BH_4_)_2_‐LiBH_4_	G1		Yes	0.4	–	94 (CV)^a)^	–	[93]
Mg(CB_11_H_12_)_2_	G4		Yes	0.25	>100 (CV)a)	99 (CV)^a)^	3.8 (Pt)	[51]
Mg[B{OC(H)(CF_3_)_2_}_4_]_2_ (Mg[B(HFIP)_4_]_2_)	G1		Yes	0.4 @0.5 mA cm^−2^	>100 @0.5 mA cm^−2^, 1 C cm^−2^	>98 @0.5 mA cm^−2^, 1 C cm^−2^	3.5 (Pt)	[55]
Mg[B{OC(H)(CF_3_)_2_}_4_]_2_ (Mg[B(HFIP)_4_]_2_)	G2		Yes	0.2 @0.5 mA cm^−2^	>100 @0.5 mA cm^−2^, 0.25 mA cm^−2^	>98 @0.5 mA cm^−2^, 0.25 mAh cm^−2^	3.5 (Pt)	[48]
Mg[B(OCH_2_CF_3_)_4_]_2_ (Mg[B(TFE)_4_]_2_)	G1		Yes	–	>200 @0.67 mA cm^−2^, 0.11 mAh cm^−2^	92.68 @0.67 mA cm^−2^, 0.11 mAh cm^−2^	–	[94]
Mg[B{OC(H)(CF_3_)_2_}_4_]_2_ (Mg[B(HFIP)_4_]_2_)	G1		Yes	–	>8000 @0.67 mA cm^−2^, 0.11 mAh cm^−2^	99.21 @0.67 mA cm^−2^, 0.11 mAh cm^−2^	4.0 (SS)	
Mg[BH{OC(CF_3_)_3_}_3_]_2_ (Mg[BH(PFTB)_3_]_2_)	G1		Yes	–	>2500 @0.67 m cm^−2^, 0.11 mAh cm^−2^	98.89 @0.67 mA cm^−2^, 0.11 mAh cm^−2^	–	
Mg[Al{OC(H)(CF_3_)_2_}_4_]_2_ (Mg[Al(HFIP)_4_]_2_)	G1		Yes	0.35 @0.5 mA cm^−2^	>500 @0.5 mA cm^−2^, 0.125 mA cm^−2^	>99.3 @0.5 mA cm^−2^, 0.125 mAh cm^−2^	3.5 (Pt)	[53]
Mg[Al{OC(H)(CF_3_)_2_}_4_]_2_ (Mg[Al(HFIP)_4_]_2_)	G2		Yes	0.06 @0.5 mA cm^−2^	>250 @0.5 mA cm^−2^, 0.25 mA cm^−2^	>99.4 @0.5 mA cm^−2^, 0.25 mAh cm^−2^	3.5 (Pt)	[48]
*Ether‐nonether dual‐solvent electrolyte*								
(*n*‐C_4_H_9_)_2_ Mg‐2(C_2_H_5_)AlCl_2_	THF	Triethylamine (10)	Yes	–	–	99 (CV)a)	1.62	[65]
		Triethylamine (20)	Yes	–	–	97 (CV)a)	1.44	
		Triethylamine (30)	Yes	–	–	89 (CV)a)	1.79	
C_2_H_5_I‐Mg	(C_2_H_5_)_2_O	Dimethyl aniline	Yes	–	–	83		[21]
MgCl_2_	THF	DPSO_2_	Yes	–	–	57.3 (CV)a)	–	[75]
Mg(TFSA)_2_	G4	EPSO_2_‐IL	Yes	–	–	50 (CV)a)	4.1 (Pt)	[15]
Mg(TFSA)_2_	G2	Isobutylamine	Yes	<0.16 @0.1 mA cm^−2^	–	90.6 @0.1 mA cm^−2^, 0.1 mAh cm^−2^	3.92 (SS) 4.03 (Ti)	[85]
Mg(TFSA)_2_	THF	DMA	Yes	0.21 @0.02 mA cm^−2^	–	56 (CV)a)	–	[23]
Mg(TFSA)_2_	G1	TMP	Yes	0.15 @0.1 mA cm^−2^	300 @0.1 mA cm^−2^, 0.025 mAh cm^−2^	78.6 (CV)a)	4.0 (Pt)	[78]
Mg(TfO)_2_	G2	TEP	Yes	0.2 @2 mA cm^−2^	>250 @2 mA cm^−2^, 4 mAh cm^−2^	99.96 @2 mA cm^−2^, 4 mAh cm^−2^	4.0 (Pt)	[80]
*(Intramolecular) Ether‐nonether hybrid electrolyte*								
Mg(TFSA)_2_	MOEA		Yes	0.08 @0.1 mA cm^−2^	>180 @0.1 mA cm^−2^, 0.1 mA cm^−2^	95.9 @0.1 mA cm^−2^, 0.1 mA cm^−2^	–	[26]
	MOPA		Yes	0.08 @0.1 mA cm^−2^	<60 @0.1 mA cm^−2^, 0.1 mA cm^−2^	<60 @0.1 mA cm^−2^, 0.1 mA cm^−2^	–	
Mg(TfO)_2_	MOEA		Yes	0.05 @0.1 mA cm^−2^	<150 @0.1 mA cm^−2^, 0.1 mA cm^−2^	94.8 @0.1 mA cm^−2^, 0.1 mA cm^−2^	–	[77]
	MOPA		Yes	0.2 @0.1 mA cm^−2^	>800 @0.1 mA cm^−2^, 0.1 mA cm^−2^	99.6 @0.1 mA cm^−2^, 0.1 mA cm^−2^	–	
*Ether‐hybrid ether dual‐solvent electrolytes*								
Mg(TFSA)_2_	G1	Bis(2‐methoxyethyl)amine	Yes	0.6 @0.1 mA cm^−2^	<50 @0.1 mA cm^−2^, 0.1 mA cm^−2^	<60 @0.1 mA cm^−2^, 0.1 mA cm^−2^	–	[24]
		MOEA	Yes	0.1 @0.1 mA cm^−2^	>100 @0.1 mA cm^−2^, 0.1 mA cm^−2^	99.5 @0.1 mA cm^−2^, 0.1 mA cm^−2^	–	
		1‐methoxy‐2‐propylamine	Yes	0.1 @0.1 mA cm^−2^	>100 @0.1 mA cm^−2^, 0.1 mA cm^−2^	99.5 @0.1 mA cm^−2^, 0.1 mA cm^−2^	3.8 (SS)	
Mg(TfO)_2_	G2	MOEA	Yes	0.1 @0.5 mA cm^−2^	>500 @0.5 mA cm^−2^, 0.25 mA cm^−2^	98.9 @0.5 mA cm^−2^, 0.25 mA cm^−2^	2.8 (Pt)	[27]
*Nonethereal electrolytes*								
MgBr_2_, Mg(SCN)_2_, Mg(ClO_4_)_2_, Mg(OC_2_H_5_)_2_, Mg(OCH_3_)_2_	Pyridine, Formamide, Benzonitrile, AN, *o*‐Toluidine, Aniline, Ethyl bromide		No	–	–	–	–	[21]
Mg(TFSA)_2_	AN		No	–	–	–	–	[81]
Mg(TFSA)_2_	GBL		No	–	–	–	–	[83]
Mg(TFSA)_2_	1,3‐Propyl diamine		Yes	0.2 @0.1 mA cm^−2^	<20 @0.1 mA cm^−2^, 0.1 mA cm^−2^	<5 @0.1 mA cm^−2^, 0.1 mA cm^−2^	–	[26]
Mg(TfO)_2_	DMF, DMA, GBL		No deposits	–	–	–	–	[82]
Mg(PF_6_)_2_	AN		No deposits	–	–	–	–	[95]
Mg[B{OC(H)(CF_3_)_2_}_4_]_2_ (Mg[B(HFIP)_4_]_2_)	Nitrile, Amide, Carbonate, Sulfoxide, Sulfite, Phosphate, Acetate, Borate, Ketone		No deposits					[17]b)
Mg(TFSA)_2_	Nitrile, Amide, Carbonate, Phosphate		No deposits					This workb)
Mg[Al{OC(H)(CF_3_)_2_}_4_]_2_ (Mg[Al(HFIP)_4_]_2_)	Nitrile, Amide, Carbonate, Phosphate		No deposits					This workb)

a) Evaluated by cyclic voltammetry.

b) As a wide variety of solvents has been evaluated in these works, only the classification of the tested solvents is listed here.

## Conflict of Interest

The authors declare no conflict of interest.
